# Performance of the cobas EZH2 mutation test on clinical samples from non-Hodgkin lymphoma patients

**DOI:** 10.1371/journal.pone.0292251

**Published:** 2023-12-14

**Authors:** Johnny Y. Shyu, Peter A. Schlag, Sylwia M. Karwowska, Chitra F. Manohar, Huan M. Truong, John W. Longshore, Guili Zhang

**Affiliations:** 1 Roche Molecular Systems, Inc., Pleasanton, California, United States of America; 2 Carolinas Pathology Group and Carolinas HealthCare System, Charlotte, North Carolina, United States of America; University of Colorado Denver, UNITED STATES

## Abstract

**Objective:**

To present the technical verification and clinical validation of the companion diagnostic assay, cobas^**®**^ EZH2 Mutation Test (cobas EZH2 Test), targeting gain-of-function *EZH2* mutations in follicular lymphoma (FL) and diffuse large B-cell lymphoma (DLBCL). The focus is on patient clinical samples proving that the test met the performance criteria required for FDA approval of a companion diagnostic test.

**Design:**

Epizyme, Inc., Eisai Co., Ltd., and Roche Molecular Systems, Inc., collaborated to develop the cobas EZH2 Test on an RT-PCR platform. The assay design needed to detect the gain-of-function *EZH2* mutations found in FL and DLBCL indications. Thus, the test was optimized for investigational purposes in a clinical trial setting. Part of its technical verification included testing of patient tumor samples with a documented diagnosis of FL and DLBCL procured from commercial vendors, and the clinical validation used patient samples from the Epizyme clinical study. Both the technical performance verification method correlation study (104 clinical commercially acquired samples) and the clinical validation accuracy study (341 patient samples from the therapeutic study) used next-generation sequencing as a reference method to establish true vs. false results by cobas EZH2 Test. The reproducibility study used a 15-member panel of DNA samples with varying *EZH2* mutation status from procured clinical FL and DLBCL patient samples under multiple variables.

**Results:**

Single and rare, infrequent double *EZH2* mutations were detected in FL and DLBCL samples. Agreements between results from cobas EZH2 and sequencing were >98% from commercial clinical samples and from the therapeutic study clinical samples.

The reproducibility study obtained 178 to 180 valid results for each panel member, with an overall invalid rate of 0.37%. The agreement for each per panel member was 100%.

**Conclusion:**

cobas EZH2 Test data demonstrated that the test is reliable and will perform well in a commercial customer environment.

## Introduction

Precision medicine treatments require parallel development of therapeutics and diagnostics, where diagnostics play a role in identifying patients targeted by the drug. Tazemetostat (TAZVERIK^®^), an epigenetic therapy for non-Hodgkin lymphoma (NHL), needed a diagnostic tool to identify mutations in the *EZH2* gene. The cobas^®^ EZH2 Mutation Test was used first as an investigational diagnostic device to screen patients for the tazemetostat clinical study to evaluate the treatment of two subtypes of NHL: follicular lymphoma (FL) and diffuse large B-cell lymphoma (DLBCL). Subsequently, the beneficial clinical outcome of the therapeutic study for patients with FL led to tazemetostat approval by the US Food and Drug Administration (FDA) in 2020 to treat adults with relapsed or refractory FL whose tumors are positive for the *EZH2* mutation and who have received at least two prior systemic therapies. It is also indicated for adults who have no other available treatment options. Contemporaneously, the cobas EZH2 Mutation Test (hereafter referred to as “cobas EZH2 Test”) was approved by the FDA as a companion diagnostic to inform treatment with tazemetostat in patients with FL [1–3].

EZH2 is the enzyme component of a multiprotein polycomb repressive complex 2, which catalyzes the methylation of lysine 27 residue of histone H3 (H3K27) [4]. Tazemetostat is a small molecule inhibitor of EZH2 histone methyltransferase activity that reduces H3K27 trimethylation (H3K27me3) levels and targets the epigenetic silencing of tumor suppressor genes and other genes involved in the development of NHL-type B-cell malignancies associated with oncogenesis [5, 6].

The observation that gain-of-function mutations in the *EZH2* gene and overexpression of EZH2 protein are frequently found in a wide variety of cancers and that those events often correlate with high-grade cancer progression and/or a poor prognosis has stimulated interest in the development of EZH2 inhibitors to use as therapeutic agents to treat cancer [7–11]. Among hematological cancers, somatic gain-of-function *EZH2* mutations are found in lymphocytes of germinal-center origin in ~8% of patients with DLBCL and in ~20–25% of patients with FL, which are two types of NHL. Mutations were identified in three hotspots in the catalytic SET domain within exons 16 and 18 at positions Y646, A682, and A692. The Y646 mutations occur as five different allelic variants: Y646F, Y646N, Y646S, Y646C, and Y646H. The other two mutations occur as A682G and A692V [12–18]. In addition to single amino acid mutations, sequencing has revealed the rare presence of double mutations (co-mutations) in less than 1% of patients [19, 20]. However, the clinical significance of co-mutations remains unknown.

The biology of B cells that bear gain-of-function mutations in EZH2 make them predisposed to respond to EZH2 inhibitors; however, EZH2 wild-type (WT) lymphomas also arise from EZH2-dependent cells and the biological response to the EZH2 inhibitors is expected to occur in these WT tumors as well. Epizyme, Inc., Eisai Co., Ltd., and Roche Molecular Systems, Inc., collaborated on the development of the cobas EZH2 Test on a real-time polymerase chain reaction (PCR) platform. The cobas EZH2 Test, similar to the cobas EGFR Test [21], is based on allele-specific PCR, a highly sensitive method to detect rare mutations in the presence of WT alleles. The test consists of two major steps: (1) a manual deoxyribonucleic acid (DNA) isolation step and (2) PCR amplification and detection of target DNA using complementary primer pairs and oligonucleotide probes labeled with fluorescent dyes to detect each mutation. Inclusion of a mutant control and a negative control in each run confirms the validity of the run. DNA allele-specific PCR is not only at least as sensitive as sequencing but can detect specific mutations in hours in WT allele tumor background, using a simple and rapid workflow. In comparison, most next-generation sequencing (NGS) methods require complex expensive workup over days for DNA library preparation, sequencing run, and extensive data analysis requiring ultra-high depth sequencing to pick up specific mutations occurring at very low frequency.

The assay design needed to fulfill the purpose of detecting the gain-of-function *EZH2* mutations found in FL and DLBCL indications. Thus, the test has been optimized for investigational purposes in a clinical trial setting. Part of its technical verification included testing of patient tumor samples with a documented diagnosis of FL and DLBCL procured from commercial vendors. The cobas EZH2 Test was used to support patient selection and cohort allocation of subjects in tazemetostat Study E7438-G000-101 in FL and DLBCL. Subsequently, patients’ clinical samples from the tazemetostat trial were available for clinical validation of the cobas EZH2 Test. In the E7438-G000-101 study, a clinically significant response was seen in the majority of patients with *EZH2* mutations and in a smaller percentage with EZH2 WT FL [2]. Ultimately, the cobas EZH2 Test was approved by the FDA (June 18, 2020) and deployed commercially as a companion diagnostic device for use in clinical practice to inform FL disease management. This paper focuses on the performance of the cobas EZH2 Test using the clinical samples that were utilized in the technical performance verification (TPV) and clinical validation of the test.

## Methods

Written informed consent was obtained from all patients participating in the reproducibility and method comparison studies. Approval from institutional Ethics Committees was obtained for all patients. The study was approved by the Institutional Review Board (IRB), WIRB-Copernicus Group IRB, 1019 39th Ave SE/Suite 120, Puyallup, WA 98374 (IRB registration number IRB00000533) and was conducted in accordance with the Declaration of Helsinki. Additional information regarding the ethical, cultural, and scientific considerations specific to inclusivity in global research is included in the S1 Checklist. Remaining studies used procured samples from third-party vendors with applicable IRB or Ethics Committee approval for sample collection. All samples were fully anonymized and data subjects were no longer identifiable for all procured samples.

### Detection of *EZH2* mutations by the cobas EZH2 test

The cobas EZH2 Test utilized manual specimen preparation to obtain DNA from formalin-fixed paraffin-embedded (FFPE) tissue as well as allele-specific PCR chemistry to detect mutations preferentially over WT sequences during real-time PCR amplification and detection. Four different reporter dyes were used to detect the EZH2 sequences targeted by the test. Amplification of the targeted EZH2 sequences were detected independently across three reactions by measuring fluorescence at the four characteristic wavelengths in dedicated optical channels. Cycle-to-threshold (Ct) values from each channel for the Mutant Control and Negative Control reactions determined if the run was valid. If the run was valid, then the Ct and relative Ct values for each sample were evaluated against acceptable ranges for each channel, as defined by analytical performance studies. The control and result validity, qualitative test results (e.g. Mutation Detected [MD], No Mutation Detected [NMD]), and mutation results in a multiplex assay setting (Y646N, A692V, Y646F, A682G, and/or Y646X [Y646H, Y646S, or Y646C]) for samples were determined automatically by the software as noted in Table 1. If *EZH2* mutations listed in Table 1 are not detected, the test result reported is “No Mutation Detected”. It was possible for more than one mutation to be present and reported by the software.

**Table 1:** Cobas EZH2 mutation test coverage.

Exon	Mutations detected	Mutation report call
**16**	Y646N	Y646N
**18**	A692V	A692V
**16**	Y646G	Y646G
**18**	A682G	A682G
**16**	Y646H, Y646S, Y646C	Y646X

Samples consisting of one 5-μm section were processed with the cobas DNA Sample Preparation Kit (Roche Diagnostics GmbH, Mannheim, Germany). The same DNA extraction method was used for samples for both PCR and sequencing testing. Detection was performed using cobas EZH2 Test kit lots; where appropriate, multiple sample preparation and amplification kit lots were utilized. The testing was performed according to the assay’s instructions for use.

### Detection of *EZH2* mutations by targeted DNA amplicon sequencing (referred to as MiSeq)

The *EZH2* mutation status was also determined in FFPE tissue using an externally validated, proprietary, targeted DNA amplicon NGS technology—considered the reference—to detect exon 16 and exon 18 mutations. NGS testing was performed by Q Squared Solutions Expression Analysis LLC (Morrisville, North Carolina). For each sample, DNA input was used to PCR amplify regions in each exon, followed by dual-indexing PCR. The sample libraries were pooled and sequenced along with positive controls, using the 2X150 bp paired-end sequencing protocol on the MiSeq platform (Illumina, San Diego, California). The limit of detection (LoD; defined as a frequency observed by at least 95% of the validation samples) for variants at loci assessed at positions that have achieved a sufficient sequencing depth of at least 2000 reads and Quality Score ≥30 for this assay was 1.5%. Specimens containing a mutant DNA sequence percentage ≥1.5% were considered *EZH2* mutation positive, whereas samples with <1.5% were considered EZH2 WT.

### Samples

For the technical verification studies, commercially available clinical tumor samples representing WT and various mutant variants of *EZH2* were acquired from 14 vendors (Analytical Biological Services, Inc.; Asterand, Inc.; Aurora Diagnostics, Inc.; Bio-Options, Inc.; BioServe, Inc.; Cambridge Biosource, LLC; Conversant Bio; Folio Biosciences; ILSbio, LLC; Indivumed GmbH; MT Group; PhenoPath, PLLC; ProteoGenex, Inc.; Tissue Source, LLC). These were de-identified remnant FFPE tumor tissue samples from biopsies. Pathology reports provided by vendor included the following: tissue type, site, pathology diagnosis, viable tumor content (%), necrosis percentage (%), surgical site (when applicable), and some demographic data. Of 104 unique FFPE tumor tissue samples, diagnoses of 29 FL and 52 DLBCL were consistent with World Health Organization nomenclature while four cases did not have a specific diagnosis but fell under the B-cell lymphoma category, one case was DLBCL/FL, and one case was nodular sclerosis. In addition, 17 samples had no final diagnosis and stayed in the “Other” category.

For the reproducibility study, genomic DNA extracted from procured clinical samples histologically confirmed as FL and DLBCL were used to build the 15-member panel representing clinically significant mutations at predetermined concentrations of 5% (LoD) and 15% (3x LoD). The panel contained WT EZH2 and EZH2 with Y646F, Y646N, Y646S, Y646C, Y646H, A682G, and A692V mutations.

For the clinical validation study, a method comparison between the cobas EZH2 Test and MiSeq sequencing evaluated a representative subpopulation of samples from DLBCL and FL from patients participating in Study E7438-G000-101. Samples were histologically confirmed as DLBCL or FL. In cases where the pathology report was not available, samples were labeled as “other.”

When available, the existing stored DNA extracted for the cobas EZH2 Test was used for sequencing; otherwise, the DNA was extracted de novo from the stored FFPE tissue sample and sequenced.

For studies where procured samples were used, samples were purchased starting in 2013 and studies were performed in 2014–2015, with the exception of the reproducibility study, which was conducted in May–July 2018. For the clinical validation study, samples from Study E7438-G000-101 were tested from June 2015–March 2020.

### Statistical analysis

#### Reproducibility study

Data were summarized by the percent agreement by panel member with the associated 95% confidence intervals (CIs). Variance component analysis was performed on Ct values from the cobas EZH2 Test for mutant panel members with a test result of MD. The mixed-effect model was used with lot, site/instrument, operator, day/run, and within-run as random effects in order to estimate between-lot, between-site/instrument (confounded), between-operator, between-day/run, and within-run effects.

#### TPV method correlation study

The positive, negative, and overall percent agreements (PPA/NPA/OPA) between the cobas EZH2 Test and MiSeq (reference method) were calculated with a 95% CI for samples with valid results obtained from both the cobas EZH2 Test and MiSeq testing.

#### Clinical validation method comparison study

The PPA and NPA between the cobas EZH2 Test and MiSeq (reference method) were calculated with 95% CIs from samples with valid results from both methods. The agreement analysis was based on overall MD or NMD results. The sample was determined as MD if one or more mutations (as covered by the cobas EZH2 Test) was detected. The adjusted agreement was calculated because different proportions of samples with MD or NMD results as determined by the cobas EZH2 Test were selected for sequence analysis. The adjusted agreements were determined by calculating the likely number of sequenced MD and NMD results that would have been found if all the patients with a cobas EZH2 Test result had undergone sequencing and obtained valid results. The 95% CIs for adjusted agreement were constructed based on the bootstrap method with 1,000 bootstrap samples. Bootstrap method was carried out by resampling from all patients with cobas EZH2 Test results. For each of 1,000 bootstrap sample, adjusted agreement estimates for PPA and NPA were obtained. The bootstrap confidence interval was constructed by estimating the 2.5^th^ and 97.5^th^ percentile of the distribution of PPA and NPA values.

## Results

### Cobas EZH2 Test internal technical performance verification studies

Before investigational use of the cobas EZH2 Test in the tazemetostat clinical study, the method was subjected to stringent verification TPV studies. Assay performance characteristics are presented in Table 2.

**Table 2:** Selected analytical characteristics of the cobas EZH2 Test.

Interference (endogenous)	• The interference of hemoglobin and triglycerides was examined in 17 NHL mutant and WT FFPE. Samples were tested with and without interfering substances. Hemoglobin and triglycerides were spiked at 2 g/L and 37 mM, respectively, according to concentrations defined by the CLSI EP7-A2 Guideline. Result: Tested levels of hemoglobin and triglycerides did not interfere with the performance of the cobas EZH2 Test.
Interference (exogenous)	• The interference of therapeutic agents for the treatment of NHL—rituximab cyclophosphamide, doxorubicin, vincristine, and prednisolone—on the performance of the cobas EZH2 Test was examined in samples from 17 mutant and WT NHL FFPE. Samples were tested in the presence and absence of drug substances at concentrations representing three times the peak serum concentration (3X Cmax) that a drug achieves after its administration or at a concentration equivalent to 3X therapeutic doses distributed in 5L of blood. • Result: Tested levels for each of the therapeutic drugs did not interfere with the performance of the cobas EZH2 Test.
Kit stability	• Real-time (2–8°C) and accelerated (37°C) stability of the reagents within the kit was assessed. • Result: Based on the results of the stability study, the data support the intended 18-month shelf life of the product.
Kit stability verification with FFPE specimen DNA	• Seven NHL FFPE samples were tested to functionally verify the real-time stability of three cobas EZH2 Test kit lots. DNA from each sample diluted to the recommended DNA input concentration was stored at ≤–20°C until the scheduled testing time point. • Result: All sample results were valid and reported the expected mutation result. Data support the intended 18-month shelf life for the product.
Slide and curl stability	• The stability of eight NHL FFPET 5-μm section samples was assessed. Samples were stored at 32°C in test tubes (i.e. as a “curl”) and when mounted on a microscope slide. • Result: All sample results were valid and reported the expected mutation result. Based on the results of the stability study, the data support the intended storage duration of up to 60 days.
Processed sample stability report	• The stability of processed specimens (extracted DNA from eight NHL FFPE samples) when stored at the following temperatures was assessed: 2–8°C, 32°C (upper limit of room temperature plus 2°C), and -20°C with multiple freeze/thaw cycles. Extracted DNA was pooled and stored at 2–8°C for up to the claimed duration of 14 days, at 32°C for up to 1 day, and at –20°C for up to 60 days with three freeze/thaw cycles. • Result: All sample results were valid and reported the expected mutation result. Based on the results of the processed sample stability study, the data support the intended claim storage durations of at least 14 days at 2–8°C, 1 day at 32°C, and 60 days at –20°C (with up to three freeze/thaw cycles).

CLSI: Clinical and Laboratory Standards Institute; cobas EZH2 Test: cobas EZH2 Mutation Test; DNA: deoxyribonucleic acid; FFPE: formalin-fixed paraffin-embedded; NHL: non-Hodgkin’s lymphoma; WT: wild-type.

### Technical performance verification, method correlation study

This study determined the estimated diagnostic accuracy of the cobas EZH2 Test by comparing results with MiSeq using commercial FFPE tissue clinical samples. A total of 104 samples (50 MD and 54 NMD as by sequencing as the reference) were used for the comparison between the cobas EZH2 Test and MiSeq (Table 3). Of the 104 specimens tested with the cobas EZH2 Test using reagent lot 1 (PL1), 50 MeSeq MD samples also tested MD by cobas EZH2 Test, and 52 of 54 MiSeq NMD samples were NMD by cobas EZH2 Test, with 98.1% (102/104) overall concordance compared with MiSeq. Using a second reagent lot (PL2), the cobas EZH2 Test results were MD for all 50 MiSeq MD specimens, and 53 of 54 sequencing NMD specimens were NMD by cobas EZH2 Test, with 99.0% (103/104) overall concordance compared with MiSeq (Table 4). Similarly, the PPA for both the PL1 and PL2 lots was 100%, while the NPA was 96.3% for PL1 and 98.1% for PL2. Two samples had discordant results between the cobas EZH2 Test and sequencing. Both samples were classified as MD positive with the cobas EZH2 Test, but the mutation levels were below the sequencing cutoff (1.5% cutoff for sequencing, compared with 0.5% for the cobas EZH2 Test). One sample was MD positive by both PL1 and PL2 (0.6% mutation level), and one sample was MD by PL1 and NMD by PL2 (0.54% mutation level).

**Table 3:** Comparison of the results between cobas EZH2 Test for PL1 and PL2 reagent lots vs. MiSeq sequencing.

		MiSeq sequencing		
		MD	NMD	Total
**cobas EZH2 Mutation Test Lot PL1**	**MD**	50	2^a^	52
	**NMD**	0	52	52
	**Total**	50	54	104
	**MD**	50	1^a^	51
**cobas EZH2 Mutation Test Lot PL2**	**NMD**	0	53	53
	**Total**	50	54	104

Where: MD = Mutation Detected, NMD = No Mutation Detected.

^a^There were two discordant samples for PL1; one sample was discordant for PL1 only and the other sample was discordant for both PL1 and PL2. For the sample that was discordant for PL2 only, the sample was retested by MiSeq NGS for confirmation. For the sample that was discordant between PL1 and PL2, the sample was retested by cobas EZH2 and MiSeq NGS for confirmation.

**Table 4:** Summary of percent agreement between cobas EZH2 Mutation Test PL1, PL2 reagent lots, and MiSeq results.

	PL1 vs. MiSeq	PL2 vs. MiSeq	PL1 vs. PL2
**Positive**	100%	100%	100%
**percent**	(50/50)	(50/50)	(51/51)
**agreement**	95% CI: 92.9%–100%	95% CI: 92.9%–100%	95% CI: 93%–100%
**Positive percent agreement >5% mutation**	100% (50/50) 95% CI: 92.9%–100%	100% (50/50) 95% CI: 92.9%–100%	100% (50/50) 95% CI: 92.9%–100%
**Negative**	96.3%	98.1%	98.1%
**percent**	(52/54)^a^	(53/54)^b^	(52/53)^c^
**agreement**	95% CI: 87.5%–99.0%	95% CI: 90.2%–99.7%	95% CI: 90.1%–99.7%
**Overall**	98.1%	99.0%	99.0%
**percent**	(102/104)	(103/104)	(103/104)
**agreement**	95% CI: 93.3%–99.5%	95% CI: 94.8%–99.8%	95% CI: 94.8%–99.8%
**Overall percent agreement >5% mutation**	98.1% (102/104) 95% CI: 93.3%–99.5%	99.0% (103/104) 95% CI: 94.8%–99.8%	99.0% (102/103) 95% CI: 94.7–99.8%

^a^Two specimens were false positive by the cobas EZH2 Mutation Test with reagent lot PL1: specimen S040 was Y646F false positive by the cobas EZH2 Mutation Test (MiSeq 0.53% Y646F); specimen S084 was Y646X false positive (MiSeq 0.054% Y646X).

^b^Specimen S040 was Y646F false positive by the cobas EZH2 Mutation Test (MiSeq 0.60% Y646F).

^c^Specimen S084 was Y646X false positive with PL1 and negative with PL2.

Of the 104 samples, three reported dual mutations by the cobas EZH2 Test, where one mutation was <5% variant allele frequency for at least one of the reagent lots. DNA eluates for both reagent lots tested by MiSeq to determine their respective mutant allelic frequencies demonstrated highly similar results (Table 5).

**Table 5:** MiSeq results for samples with double mutations reported.

Sample	Kit lot ID	Mutation result	MiSeq % mutation		
			Y646N	Y646F	Y646X
A	PL1	Y646F;Y646X	0.04^a^	24.9	4.14
	PL2	Y646F;Y646X	0.03^a^	24.6	4.44
B	PL1	Y646F;Y646X	0.01^a^	40.8	4.07
	PL2	Y646F;Y646X	0.03^a^	25.9	5.22
C	PL1	Y646N;Y646F	3.79	29.5	0.09^a^
	PL2	Y646N;Y646F	6.84	32.6	0.09^a^

PL: reagent lot.

^a^DNA sequence with mutant percentage <1.5% was considered as EZH2 wild-type.

### Reproducibility study

This study evaluated the reproducibility of the cobas EZH2 Test under factors including three reagent lots, three testing sites, two operators, and five non-consecutive days of testing. A 15-member panel of DNA samples with varying *EZH2* mutation status derived from procured clinical FL and DLBCL patient samples was used. There were 178 to 180 valid results obtained for each panel member, with a total of 180 tests per panel. Overall, the rate of invalid results was 0.37% (2690/2700). The agreement per panel member was 100%. The variance component analyses for lot, site/instrument, operator, day, and within-run were performed on the Ct value for each of the 14 mutation-positive panel members.

Within-run was a major source of variability, while other components such as site/instrument, lot, operator, and day did not contribute significantly. Overall, the coefficients of variation (CV) for total imprecision ranged from 1.5–1.9% across panel members. Within each component, the CV ranged from 0.0–1.6% across all panel members (Table 6).

**Table 6:** Coefficients of variation (CV%) for cycle threshold value of mutant panel members.

Panel member	N	Lot	Site	Operator	Day	Within-run	Total
Ex16 Y646N LoD	179	0.5%	0.4%	0.4%	1.0%	0.9%	1.5%
Ex16 Y646F LoD	179	0.3%	0.0%	0.5%	0.5%	1.3%	1.5%
Ex16 Y646H LoD	179	0.9%	0.0%	0.6%	0.4%	1.3%	1.7%
Ex16 Y646S LoD	179	0.5%	0.0%	0.5%	0.0%	1.4%	1.6%
Ex16 Y646C LoD	180	0.8%	0.0%	0.4%	0.0%	1.5%	1.8%
Ex18 A682G LoD	180	0.6%	0.0%	0.4%	0.0%	1.5%	1.7%
Ex18 A692V LoD	180	0.2%	0.0%	0.6%	1.0%	1.2%	1.7%
Ex16 Y646N 3xLoD	180	0.7%	0.0%	0.5%	0.9%	1.3%	1.8%
Ex16 Y646F 3xLoD	179	0.5%	0.3%	0.5%	0.0%	1.3%	1.5%
Ex16 Y646H 3xLoD	178	0.8%	0.0%	0.4%	0.3%	1.3%	1.6%
Ex16 Y646S 3xLoD	180	0.5%	0.0%	0.3%	0.6%	1.3%	1.6%
Ex16 Y646C 3xLoD	179	0.7%	0.0%	0.3%	0.0%	1.6%	1.8%
Ex18 A682G 3xLoD	179	0.8%	0.0%	0.4%	0.0%	1.2%	1.5%
Ex18 A692V 3xLoD	179	0.3%	0.0%	0.7%	1.0%	1.4%	1.9%

LoD: limit of detection.

### Tazemetostat clinical trial

Of the 896 patients evaluated with the cobas EZH2 Test, 889 had valid results (99%). The MD prevalence for the *EZH2* mutation was approximately 14.4% (128/889) among the patients with valid cobas EZH2 Test results. Among the MD (n = 128) results, Y646F/N/X(S/H/C) accounted for well over 90%, and A682G and A692V accounted for ~5% and 2% of the mutations, respectively. Double mutations were uncommon and detected in only four cases (Table 7).

**Table 7:** Prevalence of *EZH2* mutations by the cobas EZH2 Test in screened patients.

Mutation status	Overall n = 889, n (%)	DLBCL n = 496, n (%)	FL n = 256, n (%)	Other n = 137, n (%)
**Mutation detected**	128 (14.4%)	57 (11.49%)	58 (22.66%)	13 (9.49%)
A682G	6 (4.69%)	1 (1.75%)	5 (8.62%)	0 (0.00%)
A682G;Y646X	1 (0.78%)	0 (0.00%)	1 (1.72%)	0 (0.00%)
A692V	3 (2.34%)	1 (1.75%)	1 (1.72%)	1 (7.69%)
Y646F	40 (31.25%)	22 (38.60%)	15 (25.86%)	3 (23.08%)
Y646F;Y646X	1 (0.78%)	1 (1.75%)	0 (0.00%)	0 (0.00%)
Y646N	38 (29.69%)	18 (31.58%)	14 (24.14%)	6 (46.15%)
Y646N;Y646F	1 (0.78%)	0 (0.00%)	1 (1.72%)	0 (0.00%)
Y646N;Y646X	1 (0.78%)	0 (0.00%)	1 (1.72%)	0 (0.00%)
Y646X	37 (28.91%)	14 (24.56%)	20 (34.48%)	3 (23.08%)
**No mutation detected**	761 (85.60%)	439 (88.51%)	198 (77.34%)	124 (90.51%)

DLBCL: diffuse large B-cell lymphoma; FL: follicular lymphoma; Other: pathology result not available.

Note: The proportion for each mutation type was calculated in the population of Mutation Detected results.

### Clinical validation study

This study was performed to determine the diagnostic accuracy of the cobas EZH2 Test by comparing the results with MiSeq sequencing using samples collected from the clinical trial (NCT01897571). All subjects with an MD result and a subset of subjects with NMD (those enrolled in the clinical trial) identified by the cobas EZH2 Test were selected for sequencing. A total of 341 samples (representative of enrolled patients from clinical cohorts, non-enrolled patients with MD but who were screen fails, and enrolled patients who had invalid results) were sequenced and the results were included in the agreement analysis. The PPA was 98.3% (119/121) and the NPA was 98.6% (208/211) (Table 8).

**Table 8:** Agreement between the cobas EZH2 Test and MiSeq methods in samples from patients in clinical trial.

		NGS method (reference method)			
		MD	NMD	Invalid	Total
**cobas EZH2 Test**	MD	119	3	0	122
	NMD	2	208	3	213
	Invalid	0	3	3	6
	Total	121	214	6	341
**Agreement statistics**	PPA (95% CI)	98.3% (94.2%–99.5%)			
	NPA (95% CI)	98.6% (95.9%–99.5%)			

CI: confidence interval; MD: Mutation Detected; NGS: next-generation sequencing; NMD: No Mutation Detected; NPA: negative percent agreement; PPA: positive percent agreement.

Note: All CIs were calculated by the Wilson score method.

When selection bias adjustment was applied to account for the different rates of EZH2 MD and NMD samples selected for MiSeq testing, the prevalence-adjusted PPA and NPA was 94.7% (95% CI: 86.8%–100.0%) and 99.6% (95% CI: 99.0%–100.0%), respectively.

Among the valid EZH2 MD results from the cobas EZH2 Test and MiSeq, there were four samples with double mutations detected by both methods. In three other samples, the cobas EZH2 Test detected a single mutation, whereas MiSeq detected double mutations where one mutation was in agreement between both methods and the other was not detected by the cobas EZH2 Test (Table 9).

**Table 9:** Double mutation comparison of the cobas EZH2 Test vs. MiSeq.

cobas EZH2 Test	MiSeq				
	A682G; Y646X	Y646F;Y646X	Y646N; Y646F	Y646N;Y646X	Total
**A682G;Y646X**	1	0	0	0	1
**Y646F;Y646X**	0	1	0	0	1
**Y646N**	0	0	1	0	1
**Y646N;Y646F**	0	0	1	0	1
**Y646N;Y646X**	0	0	0	1	1
**Y646X**	0	0	0	2	2
**Total**	1	1	2	3	7

## Discussion

The cobas EZH2 Test was developed by Roche Molecular Systems, Inc. (Branchburg, New Jersey), as part of a precision medicine strategy adopted in the design of a drug study conducted by Epizyme, Inc., to assess the efficacy of tazemetostat treatment in FL and DLBCL patients, including those with mutations in the *EZH2* gene (MD, positive) as well as in those with WT status (NMD, negative) determined by the cobas EZH2 Test. Differential clinical benefit of tazemetostat treatment was demonstrated for mutant and WT EZH2 FL patients [2]. Before the cobas EZH2 Test was used for screening and patient stratification and cohort allocation in Study E7438-000-101, the performance of the cobas EZH2 Test was established using commercially available NHL patient samples. Further into the development of the diagnostic but before the drug trial was completed, the cobas EZH2 Test was validated using patient samples from Study E7438-000-101. At the time of the clinical validation, the results of the cobas EZH2 Test from patients screened in the drug trial were archived, but sequencing results were obtained from de novo MiSeq testing. Sequencing was performed either on banked extracted DNA that was used previously for molecular screening or on newly extracted DNA from the long-term stored FFPE tissue samples. Mutation coverage by both the cobas EZH2 Test and MiSeq was within codons 646, 682, and 692 of the *EZH2* gene.

In the Epizyme trial, the prevalence of *EZH2* mutations among FL and DLBCL patients was 22.7% and 11.5% in DLBCL, respectively, with Y646F (previously Y641) being the most frequent mutation detected, as measured by the cobas EZH2 Test. However, this prevalence of *EZH2* mutations in FL and DLBCL patient populations from Study E7438-000-101 differs from published data likely due to patient enrichment to fill various *EZH2* mutation-positive cohorts within the trial.

In previously published studies, patients with FL and DLBCL were molecularly tested almost exclusively with various sequencing methodologies. The point mutations at codon Y646, previously reported as Y641, (Y641F, Y641N, Y641S, and Y641H) were identified in 7% of FL and 22% of DLBCL in NHL patients in a study reported by Morin et al [12]. The proportions of *EZH2* mutations detected in FL and DLBCL patients were reversed (22% in FL and 14% in DLBCL) in a study reported by Ryan et al [14].

There are several studies that have focused on FL. Bödör et al reported Y641 mutation frequencies ranging from 17–27%, depending on the sensitivity of the sequencing method used [17]. The majority of mutations were in codon Y641 with A682 and A692 mutations detected at a lower rate. Double mutations were detected in 4 of 101 mutated patients. In a more recent study, Huet et al reported a 28% alteration in the *EZH2* gene of FL patients, with codon Y646 primarily affected as well as two rare cases of double mutations [20]. No significant difference in *EZH2* mutation frequency among FL patients was observed between Chinese and Western patients (16.9% vs. 19.6%, respectively) in a study reported by Guo et al [22].

The majority of public domain reports of *EZH2* mutation frequency in FL and DLBCL detected by various sequencing methods in multiple studies closely represent data reported from the cobas EZH2 Test in a highly curated Epizyme study. Our own method comparison study demonstrated high correlation between the cobas EZH2 Test PCR and MiSeq platforms.

The cobas EZH2 Test results and all sequenced samples by MiSeq agreed in more than 98% of cases for WT and mutation-positive *EZH2*. There were five discrepant results between the cobas EZH2 Test and MiSeq. Among discrepant samples, there was no case in which both methods detected different EZH2 variants. The sample was either an *EZH2* mutation that was missed by the cobas EZH2 Test or by the MiSeq method. This indicated that the cobas EZH2 Test might rarely generate a false-negative result. However, when the mutation was detected by the cobas EZH2 Test, the result was always confirmed by MiSeq. NGS was considered a more sensitive method with the LoD validated at 1.5%, while the cobas EZH2 Test was reliable down to 5%. However, the difference in sensitivity could not explain negative cobas EZH2 Test mutation results in samples with mutations detected by MiSeq in which the level of mutations was above 5%. There were three MiSeq negative results in samples that were mutation positive according to results from the cobas EZH2 Test. Because the NGS was assumed to be a true result, we had to consider the cobas EZH2 Test results as being false positives. However, there is a possibility that negative NGS results in those samples might be a result of compromised DNA quality due to long-term stored DNA or tissue used for de novo sequencing. Additionally, NGS also includes a PCR amplification step, and errors can occur if there are base substitutions, indels, or other alterations in the DNA sequence leading to inaccurate results. Therefore, effort was made to repeat sequencing when discordance occurred between the cobas test and NGS as described (Table 3, footnote).

Rare double mutations were detected in seven samples. In four samples, co-mutations were detected by the cobas EZH2 Test and confirmed by MiSeq; however, in three other samples, the cobas EZH2 Test detected a single mutation, whereas MiSeq detected double mutations. In those three samples, one mutation was in agreement between two methods and the other was not detected by the cobas EZH2 Test.

Both methods generated an insignificant number of invalid results. Although invalid results are to be expected in any test setting, the tests must demonstrate robust performance to minimize final unsolved results. Both the cobas EZH2 Test and MiSeq showed a low invalid rate below 3%. The high agreement between results from the PCR, the cobas EZH2 Test, and MiSeq, as well as the low invalid rate were consistent between the method correlation study run with commercial NHL samples and the method comparison study run with therapeutic trial clinical patient samples.

Similarly, the reproducibility study representing how the test will perform in a customer environment demonstrated very robust performance under all variables.

## Supporting information

S1 Checklist PLOS ONE clinical studies checklist. (DOCX)
